# Functional Architecture of Brain and Blood Transcriptome Delineate Biological Continuity Between Suicidal Ideation and Suicide

**DOI:** 10.21203/rs.3.rs-2958575/v1

**Published:** 2023-06-01

**Authors:** Shengnan Sun, Qingkun Liu, Zhaoyu Wang, Yung-yu Huang, M. Sublette, Andrew Dwork, Gorazd Rosoklija, Yongchao Ge, Hanga Galfalvy, J. John Mann, Fatemeh Haghighi

**Affiliations:** Columbia University; Columbia University; Columbia University; Columbia University; Columbia University; Icahn School of Medicine at Mount Sinai; Icahn School of Medicine at Mount Sinai; Icahn School of Medicine at Mount Sinai; Icahn School of Medicine at Mount Sinai; Icahn School of Medicine at Mount Sinai; Icahn School of Medicine at Mount Sinai

## Abstract

Human genetic studies indicate that suicidal ideation and behavior are both heritable. Most studies have examined associations between aberrant gene expression and suicide behavior, but behavior risk is linked to severity of suicidal ideation. Through a gene network approach, this study investigates how gene co-expression patterns are associated with suicidal ideation and severity using RNA-seq data in peripheral blood from 46 live participants with elevated suicidal ideation and 46 with no ideation. Associations with presence and severity of suicidal ideation were found within 18 and 3 co-expressed modules respectively (p < 0.05), not explained by severity of depression. Suicidal ideation presence and severity-related gene modules with enrichment of genes involved in defense against microbial infection, inflammation, and adaptive immune response were identified, and tested using RNA-seq data from postmortem brain that revealed gene expression differences in suicide decedents vs. non-suicides in white matter, but not gray matter. Findings support a role of brain and peripheral blood inflammation in suicide risk, showing that suicidal ideation presence and severity is associated with an inflammatory signature detectable in blood and brain, indicating a biological continuity between ideation and suicidal behavior that may underlie a common heritability.

## 1. INTRODUCTION

Between 2010 and 2020, suicide was the 12th most common cause of death in the US ^1^ . Suicidal behaviors contribute to significant health and economic burden, exceeding $70 billion per year in the United States alone (CDC, 2020). Although genetics, together with environmental and other individual factors, contribute to suicidal risk ^2^ , family and twin studies have been foundational in establishing the genetic contribution to suicidality, showing higher frequency of suicide attempt and death in monozygotic twins compared to dizygotic twins among suicide twin survivors but not non-suicide twin survivors ^3^ . Family studies suggest a significant genetic contribution with heritability ranging from 30 to 55% for suicidal ideation (SI), behavior, and death ^4 5^ , with additive genetic effect on the continuum of the phenotype spanning suicidal ideation, behavior, and death ^6^ .

Clinical studies link more severe SI, like that characterized by a plan and intent, to imminent risk of suicide attempts. Most studies have examined associations between aberrant gene expression and suicide behavior, but consideration of how such transcriptional profiles align with SI and evidence for a suicidal behavior-ideation phenotype continuum is lacking. Transcriptional studies that capture the spectrum of suicide risks using peripheral blood and brain samples can capture common elements across the molecular pathologies between ideation and suicidal behavior that may underlie a common phenotypic heritability^5, 6^. Such research may provide a genomic explanation for suicidal behavior as a risk factor for future suicidal acts^7^. To intervene effectively and efficiently, new methods are needed to identify not just who is at risk, but also to identify, using dynamic markers, times when they are at highest risk. This remains challenging, as individual vulnerability to suicide appears to be complex and multidimensional, with many contributing sociodemographic, genetic and environmental factors ^8–10^.

In recent years, there has been increasing interest in harnessing genomic technologies and large-scale genomic data to improve understanding of the underlying biology of suicide. One approach used to study the biology of suicide is to examine gene transcript expression patterns. Gene transcription are a functional output of genes expressed in a molecular pathway or process. Biological signals inducing gene transcript expression can vary in timescales from milliseconds, to seconds, hours, days, or even decades with varying transcriptional kinetics ^11^. Like the observed temporal dynamics in transcriptional activity, SI is also an inherently dynamic construct^12, 13^. As such, expression activity of genes associated with SI in comparison to expression patterns in brain of suicide decedents can provide an important snapshot of the underlying biology contributing to the intensity of suicidal state. One of the early seminal findings that resulted from genome-scale transcriptional regulatory studies in peripheral blood identified the gene *SKA2* (Spindle and Kinetochore Associated Complex Subunit 2) as a potential biomarker of suicide risk and ideation^14^ that prospectively predicted suicidal ideation and behavior in psychiatric patients^15, 16^. Subsequently, using whole genome transcript profiling via RNA-Seq, altered gene expression for spermidine/spermine N1- Acetyltransferase1 *(SAT1)* in blood also was shown to be a potential peripheral suicide risk biomarker ^14^.

However, complex phenotypes of suicide may involve interactions of multiple intertwined genes, transcription factors and gene co-expression clusters (i.e. genes with correlated levels of expression) in cells with modular but diverse functional patterns ^17^. Co-expression gene clusters represent coherent functional pathways in both normal conditions^18^ as well as in disease states^17^. Gene network approaches for identification of clusters of co-expressed genes have been applied towards discovery of genes involved in common molecular processes associated with suicidal behavior, as well as psychiatric conditions^19^. Gene network approaches capture gene-gene interactions by regarding genes as nodes and interactions as links, in which the association between each gene pair is measured by a similarity score or statistical significance of the association, whereby co-expressed clusters are then identified. Commonly used methods to generate gene co-expression networks include the Weighted Gene Co-expression Network Analysis (WGCNA)^20^ and the Multiscale Embedded Gene Expression Network Analysis (MEGENA)^21^ used in the present study. Such approaches have been widely applied in systems biology and brain research to study gene co-expression across such psychiatric and neurodegenerative diseases as depression ^22^, bipolar disorder ^23^, schizophrenia ^24^, and Alzheimer’s disease ^25, 26^.

Although gene network approaches have been applied in transcriptome studies of suicide in human postmortem brain tissue from suicide decedents^27^, to our knowledge, no study has investigated transcriptome profiles of SI in clinical samples using gene co-expression network methods to uncover biological processes and pathways underlying SI. As SI and gene transcripts are both highly dynamic, identification of gene networks associated with SI or its severity could be of utility in clinical translational studies for development of blood biomarker profiles that can be used to identify individuals at risk for suicide. To this end, for the first aim of the present study, MEGENA^21^ was used to identify co-expressed gene modules associated with SI and severity in the two weeks before the blood sample was taken. The second aim of the present study was to determine whether these SI associated co-expressed modules were also differentially expressed in gray and white matter postmortem human brain specimens of suicide decedent cases and non-suicide controls, to determine which elements of peripheral blood gene expression changes related to SI are also found in brain gene expression related to suicide death. This common pathophysiology may reflect common heritable elements between ideation and suicide or perhaps the intense suicidal ideation at the time of suicide.

## 2. METHODS

### 2.1. Samples & Subjects

*AH studies were* approved by the Institutional Review Board of the New York State Psychiatric Institute, IRB number for live subjects is #4815 and postmortem studies is 7351R.

### 2.2. Live subject sample recruitment and evaluation

Study participants were adults 20–65 years old and were recruited in the New York metropolitan area by advertising, via the Columbia University Medical Center Portal for research subject volunteers and through patient referrals from clinics and mental health professionals. Participants were evaluated at New York Psychiatric Institute. Study participants comprised three groups: 1. Healthy controls (HC, n = 27); 2. participants with a major depressive disorder (MDD) diagnosis with no history of suicide attempt (MDD/NS, n = 50); and 3. participants with MDD diagnosis with history of suicide attempt (MDD/SA, n = 23) (Table 1). All participants were evaluated using the Structured Clinical Interview for DSM-IV (SCID I) ^28^ by trained clinicians, with raters being at least Master’s level psychologists or psychiatric nurses. Axis I and II disorders were assessed using Structured Clinical Interviews for DSM-IV and Structured Clinical Interviews for DSM-IV Axis II Disorders ^29^. Healthy volunteers had no personal history of Axis I disorders, cluster B personality disorder, substance use disorder and lifetime history of suicide attempt, and had no first-degree relatives with a history of mood disorders, psychotic disorders or suicidal behavior.

Depressive symptoms were assessed by a research clinician using the 17-item Hamilton Depression Rating Scale (HAM17)^30^. Patients’ subjective perception of depression severity was assessed by means of the Beck Depression Inventory (BDI)^31^. The number, method and degree of medical damage of past suicide attempts were recorded on the Columbia Suicide History Form^32^. Suicidal ideation was measured by the Beck Scale for Suicidal ideation (SSI)^33^. Moderate to elevated SI was defined as SSI ≥ 5 corresponding to reported suicidal ideation in the last two weeks prior to the assessment, and no ideation was defined by SSI = 0.

Suicide attempt was defined as a self-injurious act with some degree of intent to end one’s life. Severity of suicide attempts was characterized by using the Suicide Intent Scale^34^, which assessed the patient’s expectation regarding the outcome of the suicidal behavior, and the Lethality Rating Scale^34^, which assessed the degree of medical injury resulting from the attempt. Psychiatric diagnoses and suicide attempts were verified in a consensus conference with research psychologists and psychiatrists. All subjects underwent physical examinations, and venous blood samples were collected in RNA Paxgene vacutainer tubes.

### 2.3. Postmortem brain samples

Brain samples were obtained from the Macedonian/New York State Psychiatric Institute Brain Collection in the Molecular Imaging and Neuropathology at the New York State Psychiatric Institute. Cause of death, excluding suicide, was determined by the medical examiner. All cases and controls were psychiatrically characterized by psychological autopsies, using a validated psychological autopsy interview method with at least one significant other ^35^. Diagnoses of major psychiatric disorder were determined using the SCID I ^36^ and suicide was determined using the Columbia Classification Algorithm for Suicide Assessment ^37^.

All suicide deaths and non-suicide deaths were sudden, without prolonged agonal state, absence of psychotropic or illegal drugs on history in the past three months confirmed by toxicological screens. Suicide decedents had a lifetime diagnosis of MDD, while sudden death controls did not have an Axis I psychiatric disorder. In total, the postmortem human brain samples included: 1. white matter cases with 9 controls i.e., non-psychiatric non-suicides who died of accidental causes and 15 suicide decedents and 2. grey matter cases with 29 controls and 21 suicide decedents (RNA-seq data for gray matter samples were downloaded from GEO database accession# GSE101521). Details on subject demographics and postmortem interval-PMI and brain pH are provided in supplemental Table S5. For tissue dissection, prefrontal cortical regions, specifically the dorsal gray matter (BA 9) and ventral white matter (BA 47) were used from frozen brain sections.

### 2.4. RNA Sequencing

For live samples, PAXgene blood tubes were thawed quickly and brought to room temperature prior to total RNA isolation according to the manufacturer’s instruction, and Globin mRNA was removed using GLOBINclear (Invitrogen/Ambion). For postmortem human brain specimens, total RNA was isolated from ~ 30–50 mg of gray or white matter tissue using RNeasy Lipid Tissue Mini Kit (Qiagen, #74804) according to the manufacturer’s instruction. All RNA samples were subject to Ribo-zero depletion, and libraries prepared using the Illumina Truseq library preparation kit and sequenced on the Illumina HiSeq 2500 (2 × 50), generating a mean of 48 million reads per sample. Reads were aligned to hg19 using STAR aligner and annotated to transcripts using Gencode v18 annotation.

### 2.5. Statistical and network analysis.

RNA-seq data for the live and postmortem samples were filtered separately, based on expression level and sample variability. Lowly-expressed genes with 0.5 count per million (CPM) in fewer than 2% of samples or with average log_2_CPM < 4 were excluded from all analyses. Variably expressed genes with log_2_CPM standard deviation ≥ 0.35 after adjustment for age and sex (via limma^38^), were included in the network analysis performed using MEGENA on the live samples only. Recommended default software parameters were used, i.e., minimum module size set to 10 and using Pearson correlation for calculation of correlation in gene co-expression in building Planar Filtered Networks (PFN)^39^. MEGENA multiscale clustering analysis was performed in the manner of hierarchical division to dissect the PFNs into coherent modules with nested clusters at various scales of resolution. Each module was then tested for association with presence of SI (SSI ≥ 5 vs. SSI = 0) and separately for ideation severity within the ideator group. Note, sample size for the low ideation group (i.e., SSI range 1-to-4, N = 8, Table 1) was limited and thus excluded from the comparison, yet still included in the MEGENA analysis.

For each module, gene expression patterns were represented by an eigengene, i.e., the first principal component of the expression levels computed using prcomp function in R. Differences in gene expression patterns between the group with moderate to elevated SI with SSI ≥ 5 (denoted as the high-SI group, n = 46) vs. the non-ideator group (SSI = 0, n = 46) was tested using the Wilcoxon rank sum test. Within the high-SI group, Spearman correlation coefficient with the eigengene expression was computed to assess association of suicidal ideation severity with gene expression patterns for each module. For modules found significantly associated with severity of SI, post-hoc analyses were also performed to test if observed associations with suicidal ideation severity’ persisted after adjusting for participants depression severity, measured by BDI and HAM17 with suicide related items within these instruments removed. These analyses were run in separate models using robust regression via lmrob function from robustbase^40^ in R, where the eigengene was set as the response, ideation severity set as the predictor and depression severity set as the covariate. In the postmortem brain RNA-seq data, age and sex adjusted log_2_CPM gene expression data were used to compute the eigengene using eigenvector coefficients obtained from MEGENA network analysis from the live samples. For comparison of module gene expression patterns between suicide decedent and non-psychiatric non-suicide cases, robust Cohen’s d (via d.robust from pysch^41^) effect sizes were computed and reported, instead of performing significance testing, due to sample size limitations.

### 2.6. Ingenuity pathway and upstream regulator analysis

All unnested modules significantly associated with suicidal ideation and severity (p < 0.05) were included in Ingenuity Pathway Analysis (IPA, QIAGEN Inc.,)^42^, using following thresholds: pathways that were significantly (Benjamini Hochberg adjusted BH p<0.1) upregulated (z-score>2) or downregulated (z-score < −2). Result of clusters with significant pathway were reported as circos plots using GOChord function from GOplot^43^ package in R. For upstream analysis and thresholds and filtering used as follows: p < 0.05, |z-score| > 2 and upstream regulators were filtered for genes, RNAs, and proteins. All *p*-values for IPA were computed using Fisher’s exact tests.

## 3. RESULTS

**Figure 1:**
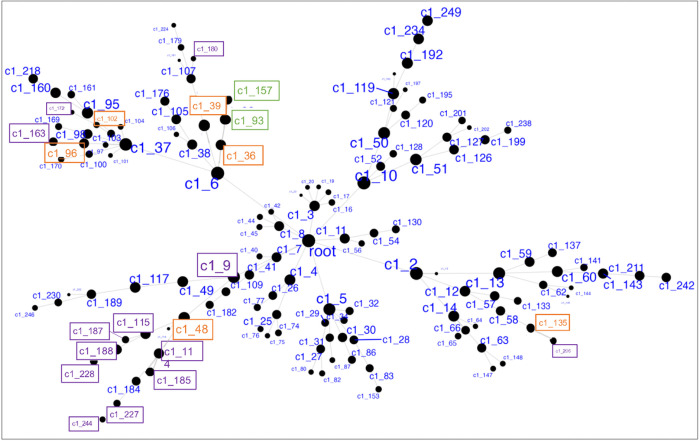
Hierarchical network structure of clusters via MEGENA. Highlighted in purple are 5 un-nested clusters (and their child clusters, total 18 clusters) showing significant differences in expression between High-SI and No-SI. In green are 2 nested clusters showing significant association with suicidal ideation severity among High SI. In orange are the 6 parent clusters.

**Figure 2:**
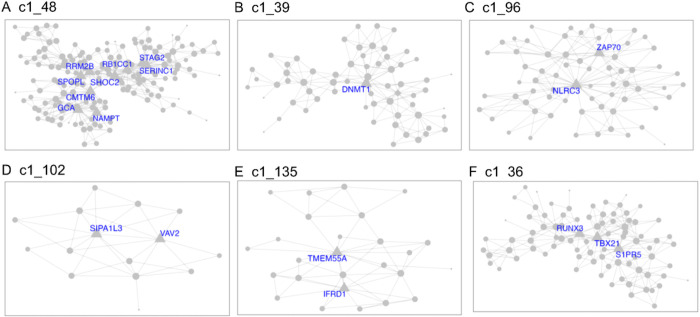
MEGENA modules identified from suicide ideation group comparisons high-SI vs. no-SI (A-E) and ideation severity analysis within the high-SI group (F), with hub genes annotated by corresponding gene symbols.

**Figure 3:**
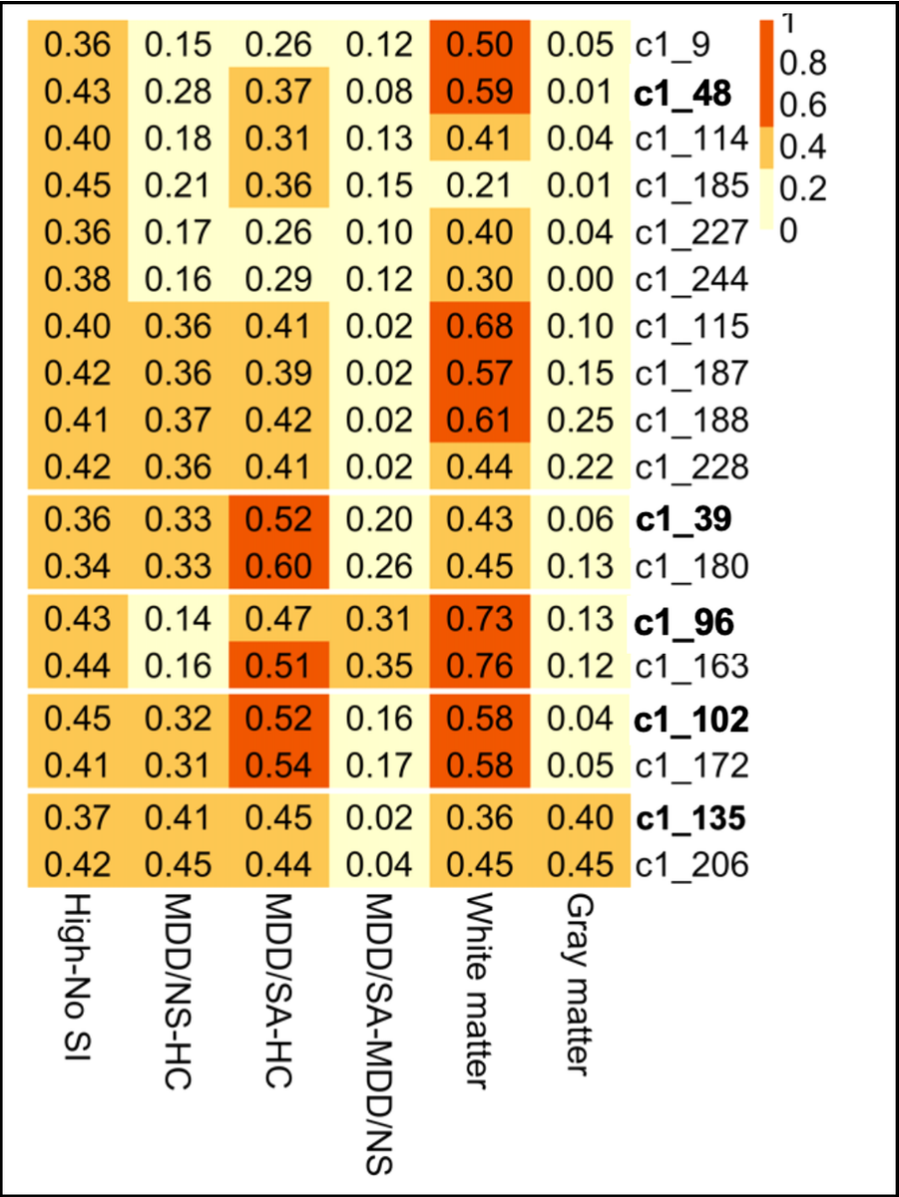
Heatmap showing effect sizes (measured as absolute values of Cohen’s D) from group comparisons of clusters’ eigengene expression, with column labels depicting various group comparisons (last two columns show white/gray matter data contrasting samples of suicide death vs. non-psychiatric non-suicides death).

Demographics and relevant clinical measurements for the N = 100 live subjects are shown in Table 1, by recruitment group. Groups did not differ by age, sex, race or ethnicity, but as expected, differed on depression and suicidal ideation scales. Of these, 92 were included in gene network MEGENA analyses: 46 participants who reported elevated suicidal ideation (denoted as high-SI with SSI ≥ 5), and 46 who endorsed no ideation (denoted as no-SI, SSI = 0), irrespective of diagnostic status. To identify gene expression signatures associated with SI, peripheral blood gene co-expression analysis was performed in live samples. Using 2740 variably expressed genes from the live samples, the MEGENA analysis produced 135 modules ranging from 10 to 607 genes per module (Fig. 1). Eigengenes representing the overall gene expression pattern of each module were computed and compared between the high-SI vs. the no-SI groups using the Wilcoxon rank sum test, resulting in 18-modules associated with SI (Table S2). Of these, 5 modules with no overlapping genes (denoted as “unnested-modules”) were identified (c1_48, c1_39, c1_96, c1_102 and c1_135, p< 0.05, see Fig. 2A–E and Table S2). Although the c1_9 module consisting of 278 genes associated with SI, this parent cluster contained two child clusters i.e, c1_48 and c1_149 modules in which the c1_49 module with 113 genes showed no association with SI (p = 0.1586); MEGENA identified the SI associated module c1_48 with superior specificity, which was then used for downstream functional analyses. To determine whether these modules’ associations with SI could be explained by diagnostic group or depression severity-related differences, post-hoc pairwise analyses were performed by comparing each module’s eigengene across the three groups (HC vs. MDD/NS vs. MDD/SA) using the Kruskal Wallis test. Two modules with significant group differences (c1_39, p = 0.0493; and its child cluster c1_180, p = 0.0458) showed significant medium sized difference between MDD/SA vs. HC (p = 0.0421, d = 0.52, Table S2 and Fig. 3) for c1_39 and trend-wise medium sized difference between MDD/SA vs. HC (p = 0.0556, d = 0.60, Table S2 and Fig. 3) for c1_180. No significant differences were observed for the remaining two comparisons for the modules c1_39 and c1_180 (p > 0.05, Table S2). Amongst the high-SI participants, 3 nested modules (Table S3) were associated with ideation severity, corresponding to modules c1_36 (r_s_ = −0.32, p = 0.0310, Fig. 2F) and corresponding child modules c1_93 (r_s_ = −0.32, p = 0.0278), and c1_157 (r_s_= 0.34, p = 0.0205). The associations between ideation severity and gene expression patterns for these three modules remained significant after adjusting for depression severity (Table S3).

Gene co-expression modules associated with suicidal ideation and severity in peripheral blood were evaluated in both gray and white matter tissue from suicide decedents and non-psychiatric non-suicide controls using whole genome transcriptome data. It should be noted that for the postmortem cases, no group difference was observed for PMI (Wilcoxon p = 0.7649), pH (Wilcoxon p = 0.9523) or RIN (t test p = 0.7916) for the white matter, and gray matter tissues (PMI: p = 0.1182, pH: Wilcoxon p = 0.3255, RIN: Wilcoxon p = 0.3515 ). Thus, following analyses were not adjusted for PMI, pH, and RIN due to limited number of postmortem cases. Also given the limited sample size of the postmortem cases, high SI associated modules from blood were evaluated using effect sizes, namely robust Cohen’s d statistic. In white matter cases, medium effect sizes were detected for 9 high-SI associated modules (Fig. 3). Specifically, amongst the five unnested-modules in white matter tissue, the effect size ranged from minimum of 0.36 for c1_135 to a maximum effect size of 0.73 for c1_96 (Fig. 3). In grey matter tissue, effect sizes ranging from negligible (d = 0.01 for c1_48) to small (d = 0.4 for c1_135) were observed for the unnested modules (Fig. 3).

**Figure 4:**
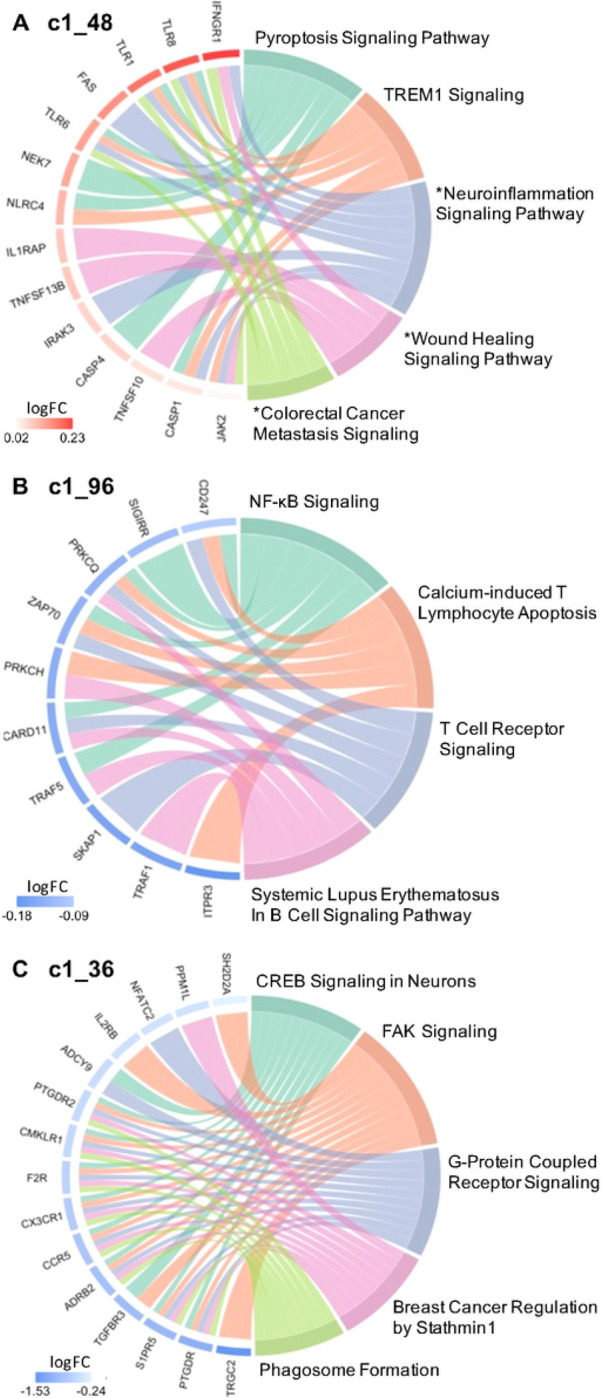
Circos plots show relationships between genes (left side) and significant IPA canonical pathways (right side). Significant IPA canonical pathways are identified for 2 SI group associated clusters (Panel A and B) and 1 suicide severity associated cluster (Panel C), based on |Z| > 2 and BH p < 0.1. Pathways marked with an asterisk (*) indicate a p-value less than uncorrected p < 0.05 but BH p >0.1. log FC = log 2 fold change expression.

Gene ontology analyses were performed to delineate the functional importance of the genes within the modules associated with high SI and SI severity, using significant threshold Benjamini Hochberg adjusted BH p< 0.1 chosen *a priori.* Pathway analysis, performed via IPA using genes from the unnested modules (Fig. 2), showed consistent gene expression differences in both blood and brain (specifically, only white matter). Notably, the high SI and high suicide related modules c1_48 and c1_96 showed enrichment of genes involved in immune and inflammatory pathways. Inflammatory pathways, including pyroptosis signaling (BH p = 0.0015, Z = 2.65), TREM1 signaling (BH p = 0.0016, Z = 2.45), and neuroinflammation pathways (trend-wise significant, BH p = 0.125, Z = 2.83), were all upregulated in the high SI associated module c1_48 (Fig. 4A, Table S4). Additionally, the high SI-and-suicide associated module c1_96 showed enrichment of genes in the NF-κB (BH p = 0.0593, Z=−2.24), T cell receptor (BH p = 0.0593, Z=−2.24), and B cell signaling immune pathways (BH p = 0.0678, Z=−2.24) (Fig. 4B, Table S4). Module c1_36, associated with SI severity showed enrichment of genes involved in cell cycle regulation i.e., CREB signaling (BH p < 0.0001, Z=−3.61), FAK signaling (BH p < 0.0001, Z=−3.87) and Stathmin regulation (BH p = 0.0001, Z=−3.46) (Fig. 4C, Table S4). No significant findings were detected in pathway analyses of genes within the c1_39 (associated with suicide attempt), and c1_102 and c1_135 modules.

## 4. DISCUSSION

Using whole genome transcriptional data from both live and postmortem samples, in the present study we investigated the associations between coordinated gene expression clusters and suicide ideation. Given the labile nature of gene expression and SI dynamics, we chose to focus on identifying transcript profiles that are associated with suicidal ideation presence and severity. Another cluster, C1_39, showed significant group difference in blood transcriptome between MDD/SA and HC groups. In addition to transcriptome data from peripheral blood from live participants, whole genome transcriptome data from gray and white matter postmortem brains of suicide decedents and controls were used for cross comparison of ideation with suicide death. Blood gene co-expression network analysis revealed a total of 18 SI associated modules transdiagnostically, and of these, 9 modules were detected in white matter with moderate effect sizes but none in gray matter.

Of the 16 hub genes (highly interconnected, Table 2) identified in these modules, 13 are novel in terms of association with suicidal ideation. Thus, they can be targeted for future studies including for consideration as potential predictors or pharmacological targets in preclinical and clinical suicide research studies. Notably among the hub genes identified, *DNMT1, RRM2B* and *GCA* have previously been shown to be associated with suicide or depression. In particular, the gene *DNMT1,* a hub gene in cluster c1_39, encodes the protein for DNA-methyltransferase 1 (DNMT1) ^44, 45^. Decreased *DNMT1* expression has been reported in the brain of suicide decedents ^46^with altered expression also detected in the limbic system and brainstem of suicide decedents as compared with controls. The second hub gene *RRM2B* codes for one of two versions of the R2 subunit of ribonucleotide reductase, which generates nucleotide precursors required for DNA replication by reducing ribonucleoside diphosphates to deoxyribonucleoside diphosphates^47, 48^. Mutation in *RRM2B* is reported to cause Autosomal-Dominant Progressive External Ophthalmoplegia with variable symptoms including depression. The RRM2B-related mitochondrial disease also leads to distinct clinical and molecular characteristics including depression^49^. The third hub gene in cluster c1_48, *GCA* (grancalcin)^50–52^ is a calcium-binding protein abundant in neutrophils and macrophages^50^, previously linked to treatment response in depression ^53^potentially through mechanisms involving innate immune processes.

Across the Sl-associated modules, gene ontology analyses demonstrated enrichment of genes involved in immune and inflammatory processes. Our findings indicate that in peripheral blood moncytes, ideators show enhanced inflammatory signatures of molecules related not only to adaptive but also to innate immune responses, such as recognition of pathogen or damage-associated molecular patterns (PAMPs/DAMPs) and activation of proinflammatory signals. A salient finding in the present study is the peripheral blood monocyte gene co-expression related to inflammatory processes in high-SI participants, with enrichment of genes in Pyroptosis Signaling, TREM1 Signaling, NF-κB signaling, T Cell Receptor Signaling, Systemic Lupus Erythematosus In B Cell Signaling, Calcium-induced T Lymphocyte Apoptosis, and trend wise enrichment of genes in neuroinflammation pathways in the high SI vs. no SI groups. This is reflected in modules c1_48 and c1_96, with a number of genes overlapping across these pathways shown in Fig. 4A and 4B. Notably in the upregulated pathways from module c1_48 (Fig. 4A), the Toll-like receptor (TLR) genes shared across these pathways in the high-SI compared to the no-SI group is consistent with prior findings, in which mRNA and protein expression of the *TLR-1* and *TLR-6* loci was higher in serum and prefrontal cortex of depressed suicide decedents compared with non-suicide decedents, respectively. Innate immune receptors such as TLRs participate in initiation of the immune activation cascade, leading to the production of cytokines in the brain. Growing evidence supports the role of these receptors in facilitating the brain to mount immune responses during systemic infection and neuronal injury^54^ as well as in mood disorders^55^. Given the role that TLRs play in cytokine production, findings that the mRNA and protein expression of TLRs are elevated in dorsolateral prefrontal cortex (dlPFC) in suicide decedents can inform understanding of upstream mechanisms in neuroinflammation that lead to abnormalities in cytokine expression in suicidal patients across the continuum of suicide risk. Although none of the individual genes identified in this study showed robust fold changes in terms of magnitude of gene expression differences between the high-SI vs. no-SI groups, they did show coordinated gene expression differences associated with suicidal ideation, in pathways previously implicated in neurodegenerative and psychiatric disorders evidenced by both animal models and human studies^56–67^.

Across the 18 Sl-associated modules found in studying peripheral blood monocytes, 9 modules were also altered in white matter with moderate effect sizes, specifically in ventral PFC white matter *postmortem* in suicide decedents. The gene ontology analyses demonstrated enrichment of genes involved in both innate and adaptive immune responses shown in modules c1_48 and c1_96 as discussed above. Since normal white matter is essential for the brain`s executive function, a greater focus on white matter integrity appears indicated for suicide research. Intact white matter is critical for functioning of prefrontal cortical areas related to attention, self-control, planning, decision-making, mood regulation and other higher cognitive abilities^68^. Loss of myelin that affects disruption of the connectivity between the PFC and the limbic system, can lead to perceptual misrepresentations, misjudgment, impulsivity, and a distorted cognitive appraisal of reality. In vivo studies of frontal lobe in depression and suicidality report reduced metabolic response^69, 70^ and disrupted brain connectivity in grey and white matter of those who attempt or die by suicide^71^. Early studies using structural magnetic resonance imaging(SMRI) also support white matter alterations in patients with history of suicide attempt by showing an increase in white matter hyperintensities^72–74^. Diffusion tensor imaging (DTI) studies have shown diminished structural integrity of white matter that provides fronto-limbic connections in suicide attempters. Decreased fractional anisotropy (FA) has been reported in ventral frontal white matter in suicide attempters, including within the region of the uncinate fasciculus that carries major ventral PFC-amygdala connections, with an association to impulsivity^75 76^. Our findings of gene modules related to suicidal ideation detected in white matter of suicide decedents may also relate to suicidal behavior, as 41% of participants with suicidal ideation in the present study had prior history of suicide attempt, as compared to 9% in the no ideation group.

Neuropathological studies also implicate white matter in suicide, with reports of increased densities of activated microglia in PFC ventral white matter of suicide postmortem cases^77^, as well as increased microglial priming and macrophage recruitment in white matter of depressed suicides^78^. Microglial activation can be stimulated through transmission of inflammatory signals from the periphery to the brain via sensory afferent projections or humoral transmigration through areas with a potentially impaired blood-brain barrier^79, 80^. In an activated state, microglia adopt different phenotypes and, in response to PAMPs or DAMPs^81^ that bind to TLR and activate NF-κB, secrete numerous proinflammatory cytokines and chemokines^82^. This observed dynamic cross-talk between peripheral inflammation and microglial-induced neuroinflammation is consistent with our findings of increased expression of inflammatory genes both in blood and in white matter, as observed in modules c1_48 and c1_96. Although prior studies employing gene network methods have primarily investigated co-expression of genes in human postmortem gray matter, with findings in line with the present study ^83–84^.

This study also identified a cluster of genes associated with suicidal ideation severity, showing coordinated expression in module c1_36 with enrichment of genes involved in cell cycle regulation including CREB signaling, FAK signaling, and Stathmin regulation (Fig. 4C and Table S4). Notably, altered cell cycle regulation previously has been linked to suicidal ideation and behavior in both peripheral blood and postmortem brain studies^85–87^. Specifically, downregulation of the gene SKA2 (spindle and KT associated 2)^88^ has been associated with depression and suicidal ideation. Expression of SKA2 is regulated by transcription factors including *CREB,* a nuclear transcription factor that also regulates transcription activity of neuronal survival and expression of different growth factors ^89^. *CREB* expression is also downregulated in multiple major psychiatric disorders, including bipolar disorder, schizophrenia, and major depressive disorder^90–92^, and decreased protein and mRNA expression of CREB is observed in postmortem brain of depressed suicide decedents ^92^. These findings are consistent with the observed downregulation of the CREB signaling pathway associated with SI severity in the present study. Further, downregulation of Stathmin-1 signaling associated with suicidal ideation severity in the present study is also implicated in fear and anxiety behaviors in animal studies. Stathmin-1 signaling is a cell-cycle regulating pathway that regulates fear and anxiety both in rodents ^93^ and humans ^94^. In one study using a social defeat mouse model of stress, knockout of *Stathmin-1* gene induced anxious hyperactivity, impaired object recognition, and decreased levels of neutral and social investigative behaviors compared to wild-type controls ^95^. Genes in the Stathmin-1 signaling pathway have additionally been implicated in psychiatric disorders ^96–99^ comorbid with suicidality.

Several strengths of the study design add confidence to the findings. The design allows comparison of genomic biology in peripheral blood related to suicidal ideation in brain to suicide death. The ideation severity findings were not attributable to depression severity. This study also has several limitations. The samples (although well-characterized through psychological assessments for live participants and psychological autopsy for postmortem cases) are small in size. Also, as is often the case in human postmortem brain studies, the measure of RNA quality RIN (RNA integrity number) can be highly variable. In this study the RIN values ranged from 2.9–9.0 for gray matter and 4.0–9.1 for white matter, with all RNAseq data passing quality control, indicating that RNA of low RIN can result in reliable RNA-seq data. This finding aligns with previous reports showing that RIN was not a sensitive measure of RNA quality for postmortem human brains^100^, since RIN is not a sensitive measure of RNA quality for substantially degraded samples, because first, the RIN score relies heavily on the amount of 18S and 28S ribosome RNAs but fails to measure the mRNA integrity directly, and second, the RIN is an overall assessment of RNA quality, and cannot serve as a specific criterion to adjust for differential RNA degradation among transcripts in downstream gene expression analyses. This limits its application in both pre-sequencing RNA sample screening and post-sequencing RNA-seq data analysis. Further, as is often the case in studies of suicidality, comorbid medical and mental health conditions are a potential confound both in study participants with SI and in the postmortem brain cases of suicide decedents and non-suicide controls used for validation of gene co-expression modules. As such, findings from this study may not generalize to other datasets with samples having varying comorbid conditions and life experiences as childhood or military trauma, etc. that may contribute to potential lack of reproducibility. Across the SI associated modules, individual genes within each module did not show significant expression differences in the SI vs. no-SI groups. This is not unexpected, since gene network approaches by design identify group(s) of genes that are changing in the same direction and magnitude, even if these changes are small. Modules of co-expressed genes thus identified in the present study, will likely be co-regulated or may belong to the same functional pathway, which need to be validated in future studies with additional experiments *in vivo* using animal models or *in vitro* using cell lines to confirm their effects on genes within relevant modules.

In conclusion, findings from this study suggest that gene expression networks are a valuable tool for identification of common pathways between suicide and psychiatric diseases, as well as factors unique to suicidality in the continuum of risk from suicidal ideation, suicidal behavior, and suicide. The SI associated co-expressed gene modules implicated with immune processes suggest potentially significant signals driving biological and molecular changes both in the periphery and in the CNS (especially in the white matter), which can potentially distinguish patients with elevated SI that may be at imminent risk for suicide. Identification of such gene expression networks occurring in both peripheral and CNS tissue as demonstrated in the present study can be integrated with other clinical measures in translational and biomarker studies of SI to improve models for suicide risk prediction.

## Figures and Tables

**Table 1:** Demographic and clinical characteristics for 100 subjects for MEGENA network building.

	Total N = 100	MDD/SA N = 23	MDD/NS N = 50	HC N = 27	P-value	Sig. pair
**Age**, Mean ± sd	36.4 ± 11.4	36.3 ± 11.1	37.5 ± 11.2	34.7 ± 12.3	0.5906	NA
**Sex**, n(%)						
Male	40 (40%)	8 (35%)	21 (42%)	11 (41%)	0.8393	NA
Female	60 (60%)	15 (65%)	29(58%)	16(59%)		
**Ethnicity**, n(%)						
Hispanic	17 (17%)	3 (13%)	8 (16%)	6 (22%)	0.7342^#^	NA
Non-Hispanic	83 (83%)	20 (87%)	42 (84%)	21 (78%)		
**Race**, n(%)						
White	59 (59%)	15 (65%)	31 (62%)	13 (48%)	0.2138^#^	NA
Black or African American	23 (23%)	5 (22%)	8 (16%)	10 (37%)		
Asian	11 (11%)	3 (13%)	7 (14%)	1 (4%)		
Other	7 (7%)	0 (0%)	4 (8%)	3 (11%)		
**Beck Scale for Suicidal ideation (SSI)**, Mean ± sd	6.7 ± 8.6	12.6 ± 9.0	7.6 ± 8.4	0±0	< 0.0001#	MDD/NS > HC, MDD/SA > HC
**Beck Depression Inventory (BDI)**, Mean ± sd	17.1 ± 14.8	27.2 ± 12.0	22.1 ± 12.6	0.9 ± 2.2	< 0.0001^#^	MDD/NS > HC, MDD/SA > HC
**Hamilton Depression Rating Scales (HAM17)**, Mean ± sd	13.6 ± 10.2	20.9 ± 5.8	19.9 ± 5.2	1.3 ± 1.5	< 0.0001^#^	MDD/NS > HC, MDD/SA > HC
**Lethality of most lethal actual suicide attempt** median [IQR]		1 [0–3]				
**Suicidal ideation group**, n*						
SSI = 0 (No-SI)	46	4	15	27		
SSI ≥ 5 (High-SI)	46	19	27	0		
0 < SSI < 5 (Low-SI)*	8	0	8	0		

* A total of 92 subjects had No or High ideation group assignment.

# non-parametric tests: Fisher’s exact for count, Kruskal-Wallis test for three group comparison with post-hoc Wilcoxon rank sum test using Bonferroni correction, and Wilcoxon rank sum test for two group comparison.

* Low-SI subjects were excluded for SI group comparison.

**Table 2:** Hub genes of clusters with differential expression pattern between high-SI and no-SI groups.

Cluster	Hub Genes (degree of intra-module connectivity)	Role of Hub Genes
C1_39	DNMT1 (17)	Gene expression regulation (DNA methylation)
C1_48	SHOC2(30),CMTM6(23),RRM2B(22),SPOPL(21),GCA(20),STAG2(19),SERINC1(18),RB1CC1(16),NAMPT(16)	Metabolism of lipid, protein, DNA and nicotinamide adenine dinucleotide (NAD); Innate immunity regulation; Cell cycle regulation; DNA repair
C1_96	NLRC3(33),ZAP70(21)	Immunity regulation (both innate and adaptive responses): cytosolic regulator of innate immunity; Motility, adhesion and cytokine expression of mature T-cells
C1_102	SIPA1L3 (11), VAV2 (9)	Cell proliferation; Endothelial cell migration Angiogenesis
C1_135	TMEM55A (15), IRFD1(11)	Metabolism of lipids; Cell proliferation

## Data Availability

The data that supports the findings of this study will be uploaded to GEO upon publication of this work.
